# Epithelial-Mesenchymal Transition and Its Regulation Mechanisms in Pancreatic Cancer

**DOI:** 10.3389/fonc.2021.646399

**Published:** 2021-04-13

**Authors:** Tuan Luu

**Affiliations:** Management & Marketing Department, Swinburne University of Technology, Hawthorn, VIC, Australia

**Keywords:** epithelial-mesenchymal transition, pancreatic cancer, metastasis, EMT-regulating factors, EMT-regulating mechanisms

## Abstract

As one of the malignancies with high mortality and high insensitivity to existing therapies, pancreatic cancer and mechanisms underlying its progression have received growing scholarly attention. The role of the epithelial-mesenchymal transition (EMT) in pancreatic cancer genesis and metastasis has been reported albeit controversy has remained. Recent insights into further EMT-regulating mechanisms underlying pancreatic cancer contribute to the nexus between EMT and this cancer type. This review will elucidate the role of EMT as a hallmark for pancreatic cancer as well as summarize EMT-regulating factors recently detected as a key advance in the research stream on EMT in pancreatic cancer.

## Introduction

In the developed world, pancreatic cancer is presently ranked the fourth among the leading causes of mortality caused by cancer diseases (1). Nonetheless, in the next few years, pancreatic cancer is becoming a cancer with the second highest mortality (2, 3). Over half of the pancreatic cancer cases have been identified at an advanced stage of the disease, which provides a partial explanation for five-year survival rate of approximately 10% (4, 5). Derived from the epithelium of the pancreatic duct, pancreatic ductal adenocarcinoma (PDAC), which occurs in over 95% of the pancreatic malignancy cases (6), is the most common pancreatic cancer subtype (7).

In undifferentiated carcinoma in some organs, epithelial-mesenchymal transition (EMT) has become a vital biological mechanism (8, 9). In the EMT process, epithelial elements undergo cytoskeleton remodelling and migratory capacity acquisition due to the loss of intercellular contacts and polarity (10). While research has reported the association of invasion and metastasis with EMT in some carcinoma types such as pancreatic cancer (11–13), some murine research works have challenged this crucial role of EMT (14). Nonetheless, EMT has been reported to contribute to pancreatic cancer cells’ drug resistance (14) as well as relate strongly to poor prognosis of PDAC (15). Moreover, recent evidence has been lent to genetic, molecular, and biochemical mechanisms mediating the EMT process in progression and metastasis of pancreatic cancer (16, 17), which strengthens the view of EMT as a cancer hallmark. While prior reviews have largely focused on molecular (e.g., Elaskalani et al. (18), Safa (19)) or biochemical mechanisms (e.g., inflammation, Khalafalla and Khan (20), Wang et al. (21)) underlying the EMT process in pancreatic cancer, this essay depicts recent evidence for the role of EMT-regulating mechanisms in pancreatic cancer in terms of genetic, molecular, and biochemical aspects. This review summarizes the findings published mainly in 2020 using EMT and pancreatic cancer as the two keywords for searching relevant articles. The remaining of the paper portrays EMT genetic and molecular mechanisms and EMT regulation mechanisms in pancreatic cancer, and concludes with the discussion on how they may direct clinical practice and future research.

## The Role of EMT in the Biology of Pancreatic Cancer

As a morphologic cellular program, EMT refers to an epithelial-to-mesenchymal state transition, whereas epithelial cells undergo phenotypic and genotypic transformations to obtain mesenchymal phenotype (21). While the epithelial phenotype is viewed as colonizable and stable, the mesenchymal phenotype is deemed to be capable of resistance to apoptosis, invasiveness, and migratory capacity (21).

**Figure 1:**
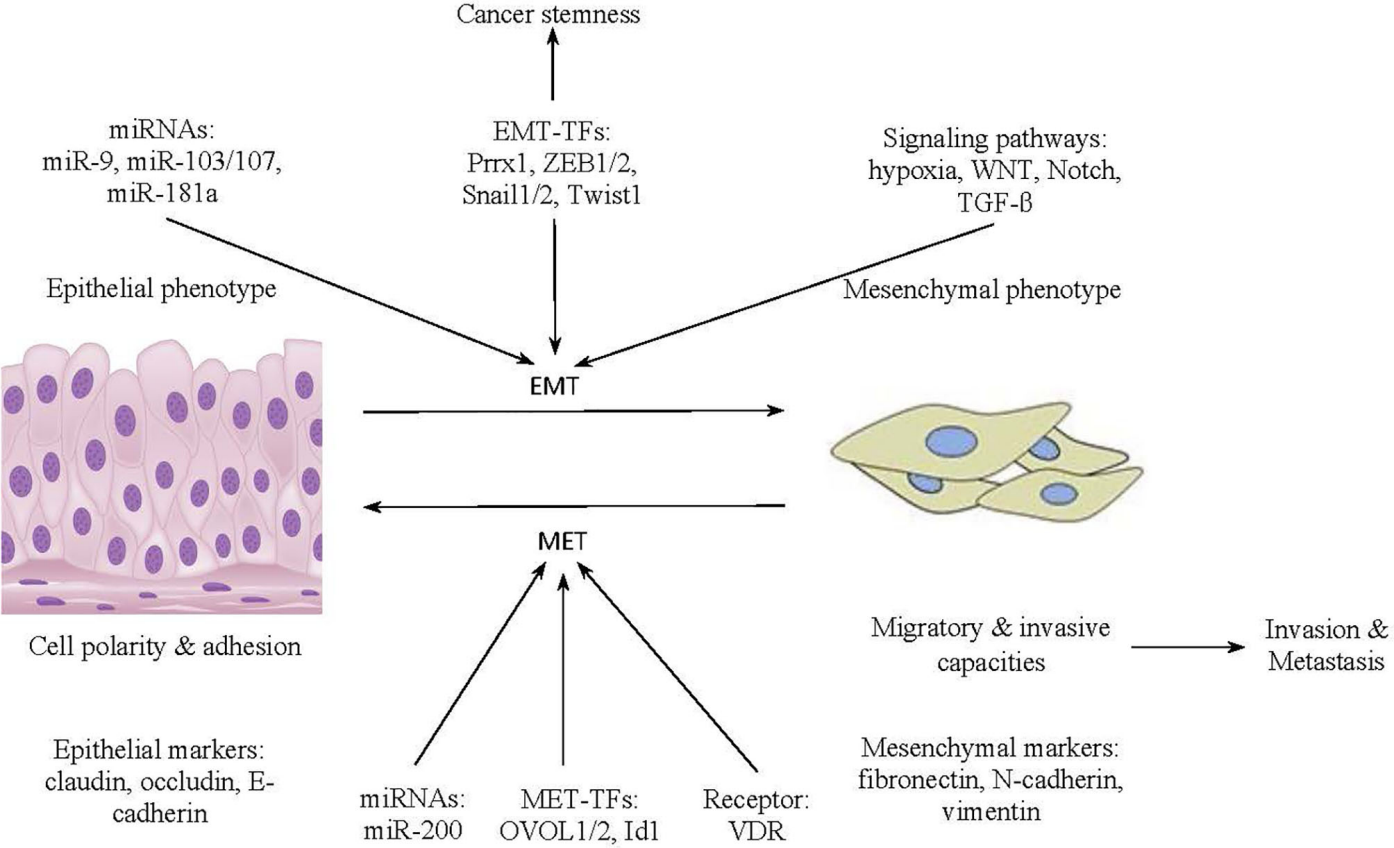
The transformation from the epithelial to the mesenchymal phenotype in cancer cells.

In diverse tissues in the body, epithelial sheets maintain their structural integrity thanks to epithelial cadherin molecules known as cell surface E-cadherin (22). E-cadherin molecules contribute to lateral junctions between epithelial cells with apical–basal polarity (22). As displayed in **Figure 1**, the transformation of cancer cells from an epithelial phenotype to a mesenchymal phenotype is activated by the expression of miRNAs (e.g., miR-9, miR-103/107, miR-181a) and EMT transcription factors (EMT-TFs) (e.g., Prrx1, ZEB1/2, Snail1/2, Twist1), as well as the triggering of signaling pathways (e.g., hypoxia, WNT, Notch, TGF-β) (23). Moreover, the transformation occurs in tandem with expression of mesenchymal markers and the suppression of epithelial markers (23). Specifically, undergoing morphological modifications from polygonal shapes to spindle shapes, transformed mesenchymal cells accumulate markers, comprising fibronectin, N-cadherin, and vimentin, as well as demonstrate loss of E-cadherin-mediated cell adhesion (24). Furthermore, once an EMT program is activated, cancer cells acquire migratory and invasive capacities that facilitate cancer invasion and metastasis. Cancerous cells also accrue the stem-like attributes under the influence of EMT transcription factors. Reaching the metastatic sites, the mesenchymal cells in pancreatic cancer undergo the MET process to reverse back to the epithelial phenotype for cancer colonization. The MET process is activated by receptor (VDR), miRNAs (e.g., miR-200), and transcriptional factors (e.g., OVOL1/2, Id1) (23).

Due to its role in leveraging extracellular matrix component production, resistance to apoptosis, and migratory capacity, EMT has been reported to be a crucial element in carcinoma progression (10, 22). Pathological analyses of surgically resected specimens of pancreatic cancer has demonstrated EMT-related molecules (25). Cell infiltration in pancreatic cancer has been found to be related to increased expression of vimentin and diminished E-cadherin (26, 27). EMT features have been exhibited in a mouse model of invasive pancreatic carcinoma cells (24). These pathological analyses indicate the role of EMT as a vital biochemical mechanism in progression of carcinomas in general and pancreatic cancer in particular.

## EMT Genetic and Molecular Mechanisms in Pancreatic Cancer Progression and Metastasis

### The Role of EMT in Carcinoma Progression

In various carcinoma types including pancreatic cancer, stemness acquisition can be induced by EMT activation (22, 28). Compared to tumors without cancer stem cells, tumors that have a subpopulation of cancer stem cells display greater expression of cell surface markers such as CD24low and CD44high (29). Furthermore, by self-renewing, cancer stem cells can self-differentiate into tumorous cells (22). Specifically, the pancreatic cancer stem cell surface marker c-Met reacts to secreted ligands and markers CD44 and CD24 foster intercellular interactions, thereby activating pathways such as Stat3, Notch, and β-catenin in pancreatic cancer stem cells and thus stimulating self-renewal (30).

Furthermore, in early-stage carcinomas, tumorous cells pathogenetically exhibit a more epithelial like state. However, through acquiring mesenchymal state markers (e.g., neural cadherin) and losing epithelial markers (e.g., E-cadherin), tumorous cells gradually develop more mesenchymal state in carcinoma progression (22, 26).

### EMT in Metastasis of Pancreatic Cancer Cells

In vitro analysis has demonstrated that invasive traits are exhibited in pancreatic intraepithelial neoplastic cells that involve in the EMT process (27). Different combinations of EMT-TFs contribute to phenotypic change during the EMT when the epigenome of invasive tumor cells undergo deacetylation and demethylation processes. DNA methylation and histone demethylase mediate the expression of miR-200 family (31). Histone deacetylase 1 (HDAC1) and HDAC2 are recruited by ZEB1 (32). Nevertheless, pancreatic cancer metastasis is driven by complementary subfunctions of different EMT-TFs (12). For instance, due to its strong effects on phenotypic plasticity and colonization capacity of pancreatic tumor cells, EMT-TF Zeb1 drives pancreatic tumour progression to late-stage metastasis, in contrast to no effects found for the EMT-TFs Twist and Snail on pancreatic cancer cell metastasis (12).

Circulating cancerous cells, through the EMT process, play a crucial role in dissemination and colonization (17). EMT may involve in this dissemination process of cancerous cells since epithelial and mesenchymal attributes can be found in most circulating tumor cells (33, 34). In the primary tumor, cancerous cells undergo EMT to develop into circulating cancerous cells in the microenvironment rich in TGF-β-associated platelets (21). Circulating EMT phenotypes may also develop into circulating cancerous cells (21). Mesenchymal markers in circulating cancerous cells reflect their ability to colonize distant organs (35). However, after extravasating and circulating in the bloodstream, epithelial cancerous cells can also approach distant organs (21). This is indicative of the salience of EMT/MET processes in secondary tumorigenesis of epithelial cancerous cells (35).

## Advances in EMT Regulation Mechanisms in Pancreatic Cancer

A key advance in the stream of research on EMT in carcinoma in general and pancreatic cancer in particular is the identification of further regulating mechanisms mediating EMT process in progression and metastatic activity of pancreatic tumor cells. This identification in recent studies on pancreatic cancer (see the summary of the publications mainly in 2020 in **Table 1**) is a key advance due to the light it has shed on the controversy on EMT role in carcinoma progression and metastasis (14).

**Table 1:** EMT regulation factors involved in pancreatic cancer progression and metastasis.

EMT regulation factors	Underlying mechanisms	References
BACH1	plays a critical role in PDAC malignant progression through regulating EMT process.	(36)
CCDC80	represses EMT markers’ expression.	(16)
ID1	uncouples EMT from apoptotic activity.	(37)
L1CAM	contributes to stemness in EMT.	(38)
Circulating tumor cells	react to EMT-inducing signals from the tumor microenvironment and accrue mesenchymal characteristics.	(39, 40)
Rab27a GTPase	triggers EMT processes once downregulated.	(17)
ENO2	induces EMT once deacetylated.	(41)
EMT proteins	involves in miR-548t-5p’s inhibitory effects on metastatic activity.	(42)
HMGA2	suppresses E-cadherin and leveraged b-catenin expression.	(43)
TMEM158	activates EMT once upregulated.	(44)
lncRNAs H19	stimulates EMT process through antagonization of let-7.	(45)

First, recent research in this stream has lent credence to genetic regulating mechanisms in relation to EMT process. Sato et al. (36) investigated the role of BTB and CNC homology 1 (BACH1) in the genesis of pancreatic ductal adenocarcinoma (PDAC). Their findings indicate that through regulating the EMT process, downstream genes (e.g., CDH1) and BACH1 play a critical role in PDAC malignant progression and prognosis. Another work by Hong et al. (16) delved into the role that CCDC80, a tumour suppressive gene, plays in pancreatic cancerous cells’ EMT process. The authors revealed that EMT markers’ expression, formation of colony, and migration are suppressed by CCDC80’s ectopic expression.

Second, this research stream has further unravelled molecular regulating mechanisms underlying EMT in carcinoma progression. A recent study by Huang et al. (37) on PDAC revealed the role of ID1 in uncoupling EMT from apoptotic activity. Cave et al. (38) further looked at the molecular mechanism regulating stemness in EMT in pancreatic stellate cells in PDAC. The results demonstrated the tumor-suppressing function of L1 cell adhesion molecule (L1CAM), which contributes to stemness in EMT. The results further revealed that through TGF-β-Smad2/3 signalling, pancreatic stellate cells’ TGF-β1 exerts a negative impact on expression of L1CAM and in turn stemness in EMT. Gemenetzis et al. (39) found the potential of epithelial circulating tumor cells (CTCs) (eCTCs) and epithelial/mesenchymal CTCs (mCTCs) as molecular biomarkers of pancreatic cancer status. Epithelial cells react to EMT-inducing signals from the tumor microenvironment and accrue mesenchymal characteristics and, in turn, tumor-initiating potential. Their findings indicated that preoperative CTCs counts were the sole predictors of early recurrence within 12 months from surgical resection in post-neoadjuvant and chemo-naive patients. White et al. (40) further reported the association of portal vein blood CTC numbers with overall survival of PDAC.

Third, some works in this research strain aimed to unfold biochemical mechanisms regulating EMT in invasion and metastasis of pancreatic cancer cells. In their recent work, Kren et al. (17) reported that through downregulating Rab27a GTPase, biogenesis of disrupted extracellular vesicles in pancreatic cancerous cells may trigger EMT processes, which in turn enhance tumorous invasion as well as colonization of distant organs. Delving into PDAC metastasis, Zheng et al.’s (41) study unveiled that glycolytic enzyme Enolase 2 (ENO2), once deacetylated, can induce EMT in cells in patients with PDAC, thereby promoting metastasis of PDAC cells. Ge et al.’s (42) findings indicate the involvement of EMT proteins in miR-548t-5p’s inhibitory effects on metastatic activity of pancreatic cancerous cells. Yang et al.’s (43) study on pancreatic cancer metastasis revealed that in Bxpc-3 and Mia PaCa-2 cells, high mobility group AT-hook 2 (HMGA2) suppressed E-cadherin and leveraged b-catenin expression. These results indicate that pancreatic cancer metastasis may be promoted by HMGA2 *via* activating EMT processes.

Fourth, EMT research stream in pancreatic cancer has also delved into mechanisms mediating EMT processes for both progression and metastasis of pancreatic cancerous cells. Investigating the functioning of transmembrane protein 158 (TMEM158) in pancreatic cancer, Fu et al. (44) have found that TMEM158, once upregulated, not only stimulates cancer progression but likewise activates EMT and thereby executes its metastasis-inducing role. Furthermore, Wang et al. (45) have studied long noncoding RNAs (lncRNAs) H19 in PDAC cells and found that, through antagonization of let-7, H19 stimulates the EMT process and thereby promote PDAC cell progression and migration.

In a nutshell, our review focuses on recent advancements on identification of EMT regulation factors in genetic, molecular, and biochemical aspects including BACH1, CCDC80, ID1, L1CAM, CTCs, Rab27a GTPase, ENO2, EMT proteins, HMGA2, TMEM158, and lncRNAs H19. It distinguishes itself from prior reviews in relation to mechanisms underlying EMT processes. For instance, a review by Elaskalani et al. (18) focused on the role of loss of E-cadherin-mediated cell adhesion in creating an elongated mesenchymal phenotype in invasion and metastasis of pancreatic cancer. Safa’s (19) review discussed molecular mechanisms underlying the behaviours of cancer stem cells in PDAC with a focus on PCSC markers Tspan8, alpha6beta4, CD44v6, CXCR4, LRP5/6, LRG5, claudin, EpCAM, and CD133. Wang et al. (21) reviewed the role of the inflammation in induction of EMT as well as the role of cancer stem cells in the tumorigenesis, colonization, and metastatic processes in pancreatic cancer. Khalafalla and Khan’s (20) review discussed the role of the inflammation environment in promoting EMT and the key pro-inflammatory signaling pathways involved in PDAC pathogenesis.

## EMT-Regulating Mechanisms for Clinical Practice and Future Research

### Clinical Implications of EMT-Regulating Mechanisms

Recent findings on the role of mechanisms regulating EMT processes in pancreatic cancer provide diagnostic, prognostic, and therapeutic implications for patients with this disease. EMT regulating mechanisms demonstrate diagnostic and prognostic values. Recent analyses have revealed that some mechanisms that regulate EMT processes function as strong predictors for outcome or therapy response among patients with pancreatic cancer (36, 38). For instance, high expression of BACH1 that regulate EMT is linked with poor prognosis of pancreatic cancer (36). The finding on tumor-suppressing role of L1CAM in reversing stemness in the EMT activation process offers prognostic value since restoration of L1CAM expression contributes to sensitizing pancreatic cancer cells to chemotherapy and in turn enhancing prognosis for patients with PDAC (38). The finding with reference to increased numbers of CTCs especially ones with mesenchymal traits as predictors of PDAC recurrence demonstrates the role of CTCs as a molecular biomarker of progression of pancreatic cancer disease and response to therapy (39). Portal vein blood CTC counts further serve as an indicator for PDAC overall survival (40).

The empirical association between TMEM158 overexpression and pancreatic cancer cell progression *via* EMT stimulation indicates that TMEM158 can serve as a prognostic indicator for development of pancreatic tumor in terms of blood vessel invasion, TNM stage, and tumor size (44).

EMT regulating mechanisms imply potential therapeutic strategies for pancreatic cancer patients. For instance, understanding the role of CCDC80 in repressing EMT markers and consequently pancreatic cancer cell invasion and migration indicates the value of vactosertib-nal-IRI/5-FU/LV combination, which demonstrates a higher variance in activating CCDC80 and further repressing EMT markers than the monotherapy with vactosertib. Furthermore, the finding on the function of deacetylated ENO2 in fostering PDAC metastasis *via* inducing EMT represents a potential strategy to control metastasis of PDAC cells through the use of IGF-1R inhibitors (e.g., Linsitinib) to block IGF-1-induced deacetylated ENO2 (41). A recent finding on metastasis inducing function of TMEM158 through EMT activation (44) also indicates this tumor promoter should be a target for pancreatic cancer therapy.

### Implications for Future Research

Our review of recent pancreatic cancer studies has revealed a focus on CDH1 and CCDC80 as genes that suppress EMT markers or impact EMT processes in pancreatic cancer progression. An extension of this research stream should be to investigate the role of other tumor suppressing genes such as PTEN and CDKN2A (46) as genetic regulating factors underlying EMT process in proliferation of pancreatic cancer. In addition, the suppressing role of CCDC80 in EMT processes should be examined in other carcinoma types than pancreatic (16), hepatocellular (47), and lung carcinoma (48).

From the role of CTCs as a biomarker for PDAC recurrence, future studies should be extended to the systemic aspect of PDAC in the form of not only CTCs but disseminated tumor cells (DTCs) as well. The link between portal vein blood CTC counts and PDAC overall survival suggests further studies on selective omission of adjuvant chemotherapy for patients treated preoperatively and tailored surveillance intensity for patients without portal vein blood CTCs at PDAC resection (40).

This review further demonstrates a focus only on the tumor suppressor role of L1CAM out of the adhesion molecule family members. This suggests that further investigations should be conducted into how other adhesion molecule family members such as ALCAM and NCAM (49) relate to EMT and stem cancer cells in pancreatic cancer. Additionally, this role of adhesion molecule family members should be further studied on patients with carcinoma types other than pancreatic (38) and colorectal cancer (50).

Prior research has revolved around the relevance of glycolytic enzyme Enolase 2 (ENO2) to EMT processes in pancreatic cancer (41). Nevertheless, by virtue of potential effects of other glycolytic enzymes such as pyruvate kinase M2 (PKM2), glyceraldehyde-3-phosphate dehydrogenase (GAPDH), and triose phosphate isomerase (TPI) on EMT in cancer cell metabolism (51), future research on pancreatic cancer as well as other carcinomas should investigate their glycosylated forms and effects of these forms on pancreatic cancer progression. Furthermore, future research should look into the metastasis inducing role of transmembrane proteins other than TMEM158 such as claudins, occludins, and MARVEL-domain proteins in relation to EMT activation (52).

## Conclusion

Survival rate of patients with pancreatic cancer, especially PDAC, has not notably improved despite considerable research efforts (17). Regardless of the debate on the link of the EMT process with pancreatic cancer colonization and migration (13), recent studies on EMT regulation factors and mechanisms have cast some new light on the role of these mechanisms in pancreatic cancer progression, invasion, migration, and prognosis. Recent evidence has lent credence to the link of pancreatic cancer cell dissemination to EMT regulation factors such as deacetylated glycolytic enzymes (e.g., ENO2) (38) or EMT proteins in miR-548t-5p (39). Research has further identified more EMT regulation factors that have involved in pancreatic cancerous cell progression such as BACH1, CCDC80, L1CAM, CTCs, and TMEM158 (15, 35, 37, 41). Some EMT-regulating factors such as L1CAM and TMEM158 function as strong prognostic indicators (37, 41), while some other factors such as CCDC80, ENO2, or TMEM158 appear as new therapeutic angles for controlling invasion and migration of pancreatic cancerous (15, 38, 41). Further EMT regulation factors and mechanisms should be explored as extensions of existing studies in the field, as well as further translation of recent evidence on these mechanisms into clinical practice is needed to enhance pancreatic cancer survival rate.

## Author Contributions

The author confirms being the sole contributor of this work and has approved it for publication.

## Conflict of Interest

The author declares that the research was conducted in the absence of any commercial or financial relationships that could be construed as a potential conflict of interest.
